# Supplementary material for: A kinetic model predicts SpCas9 activity, improves off-target classification, and reveals the physical basis of targeting fidelity
Source: Nat Commun. 2022 Mar 15;13:1367. doi: 10.1038/s41467-022-28994-2 (PMC8924176; doi:10.1038/s41467-022-28994-2)
Supplement: Supplementary file 1 — Supplementary Information [file 41467_2022_28994_MOESM1_ESM.pdf]

Supplementary Information for

A kinetic model predicts *Sp*Cas9 activity, improves off-target classification, and reveals the physical basis of targeting fidelity

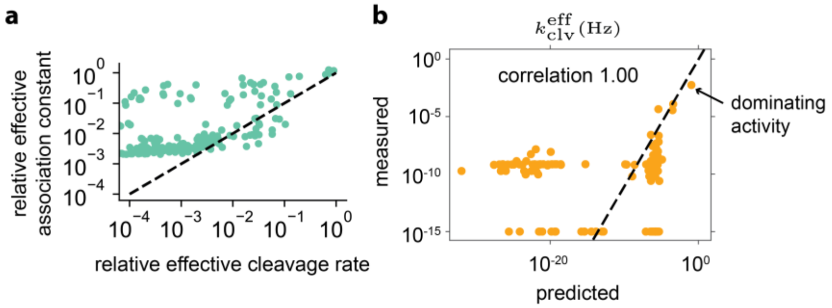

Supplementary Figure 1 a) Correlation plot of measured effective cleavage rates (NucleaSeq) relative to on-target, versus effective association constants (CHAMP) relative to on-target. The dashed line indicates where these relative measures would be equal. **b)** Correlation plot of effective cleavage rate measurements (NucleaSeq) versus model predictions for highly mismatched off-targets (three or more mismatches). The model successfully separates out the single target that dominates the Pearson-correlation calculation, resulting in a perfect Pearson correlation. The dashed line represent perfect quantitative prediction.

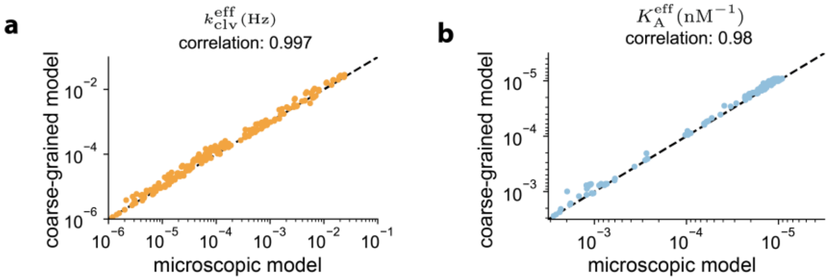

Supplementary Figure 2 a) Correlation between predictions for effective cleavage rate using the microscopic kinetic model (**Figure 1a**) and the coarse-grained kinetic model (**Figure 5a**). **b)** Same as **a**, for effective association constants. The very high correlation for both quantities shown that the coarse grained model retains the predictive power of the microscopic model. The dashed line represent perfect quantitative correspondence between models.

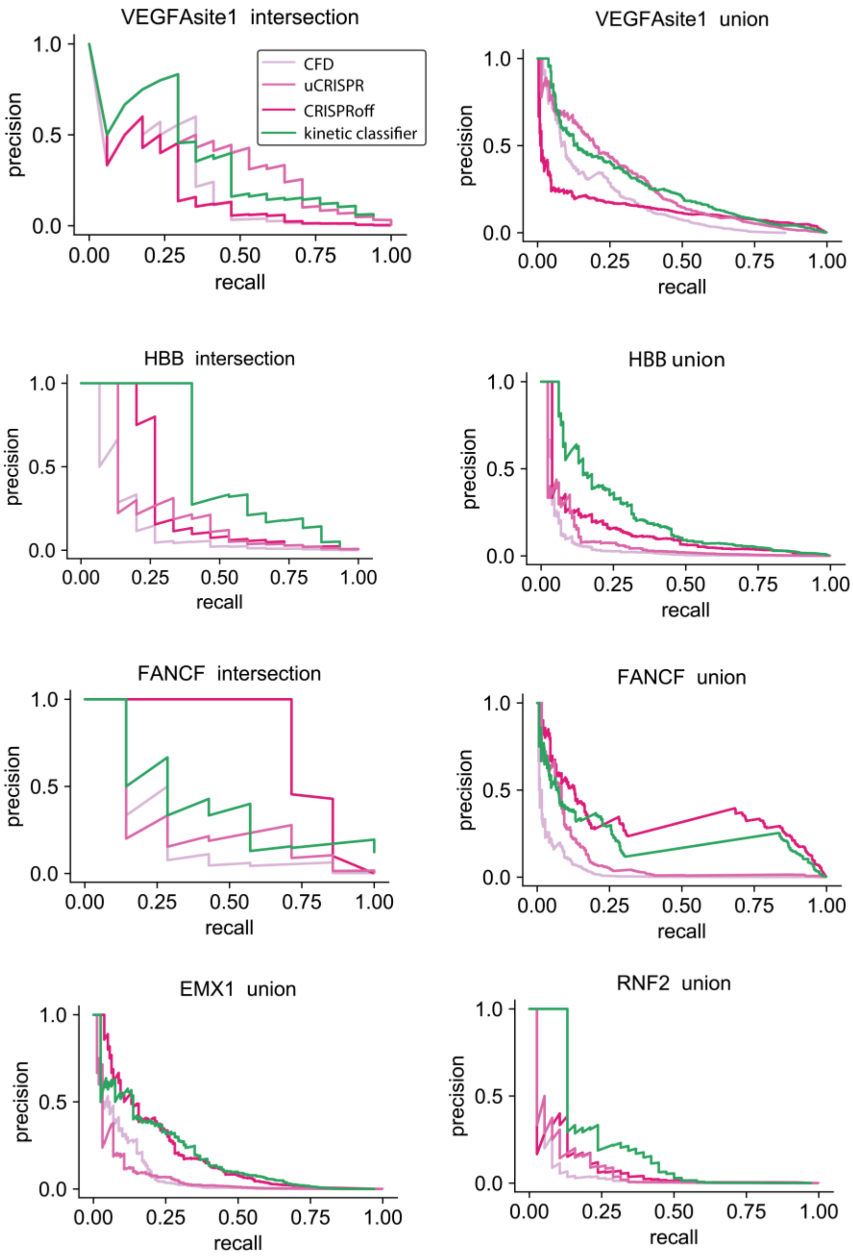

Supplementary Figure 3 a) Precision-recall curves for the CFD score (light purple), uCRISPR score (purple), CRISPRoff (dark purple), and our kinetic classifier (green) for the FANCF, VEGFA site 1, EMX1, HBB and RNF2 target sites. We use union to denote curves where all experimentally identified off-targets are used as true positives, and intersection to denote curves where off-targets identified in all experiments are used as true positives.

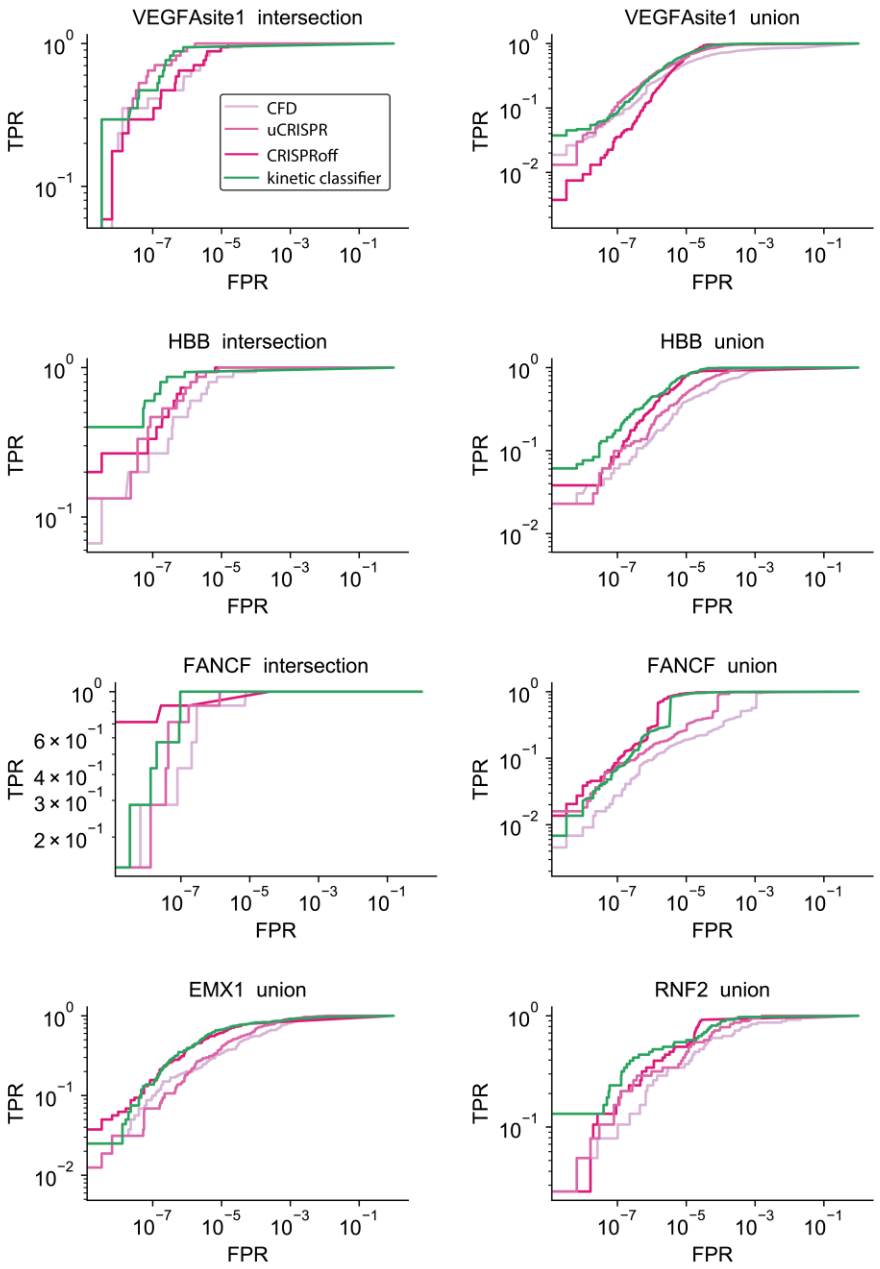

Supplementary Figure 4. Receiver-operator characteristic curves for the CFD score (light purple), uCRISPR score (purple), CRISPRoff (dark purple), and our kinetic classifier (green) for the FANCF, VEGFA site 1, EMX1, HBB and RNF2 target sites. We use union to denote curves where all experimentally identified off-targets are used as true positives, and intersection to denote curves where off-targets identified in all experiments are used as true positives. Our data sets are unbalanced, with many more true negatives than true positives. This results in a true-positive rate (TPR) that increases very rapidly with the false-positive rate (FPR) (note the log-scale used). This renders the area under the curve very close to one in every case, and to allow us to differentiate between models we instead choose to base our analysis in precision-recall curve (**Supplementary Figure 3**).

target	Technique and reference	guide sequence
EMX1	BLESS[1]	GAGTCCGAGCAGAAGAAGAAGGG
	BLISS[2]	GAGTCCGAGCAGAAGAAGAAGGG
	CIRCLE-seq[3]	GAGTCCGAGCAGAAGAAGAANGG
	Digenome-seq[4,5]	GAGTCCGAGCAGAAGAAGAAGGG
	GUIDE-seq[6]	GAGTCCGAGCAGAAGAAGAANGG
	HTGTS[7]	GAGTCCGAGCAGAAGAAGAAGGG
FANCF	CIRCLE-seq[3]	GGAATCCCTTCTGCAGCACCNGG
	Digenome-seq[4,5]	GGAATCCCTTCTGCAGCACCTGG
	GUIDE-seq[6]	GGAATCCCTTCTGCAGCACCNGG
	SITE-seq[8]	GGAATCCCTTCTGCAGCACCTGG
HBB	CIRCLE-seq[3]	GTTGCCCCACAGGGCAGTAANGG
	Digenome-seq[4,5]	CTTGCCCCACAGGGCAGTAACGG
RNF2	CIRCLE-seq[3]	GTCATCTTAGTCATTACCTGNGG
	Digenome-seq[4,5]	GTCATCTTAGTCATTACCTGAGG
	GUIDE-seq[6]	GTCATCTTAGTCATTACCTGNGG
VEGFA_site1	CIRCLE-seq[3]	GGGTGGGGGGAGTTTGCTCCNGG
	Digenome-seq[4,5]	GGGTGGGGGGAGTTTGCTCCAGG
	GUIDE-seq[6]	GGGTGGGGGGAGTTTGCTCCNGG
	HTGTS[7]	GGGTGGGGGGAGTTTGCTCCTGG
	SITE-seq[8]	GGGTGGGGGGAGTTTGCTCCTGG

Supplementary Table 1: Data sets used to collect true positives for whole genome analysis.

Supplementary references

1. Slaymaker, I. M. *et al.* Rationally engineered Cas9 nucleases with improved specificity. *Science (80-.).* **351**, 84–88 (2016).
2. Yan, W. X. *et al.* BLISS is a versatile and quantitative method for genome-wide profiling of DNA double-strand breaks. *Nat. Commun.* **8**, 1–9 (2017).
3. Tsai, S. Q. *et al.* CIRCLE-seq: A highly sensitive in vitro screen for genome-wide CRISPR-Cas9 nuclease off-targets. *Nat. Methods* **14**, 607–614 (2017).
4. Kim, D. *et al.* Digenome-seq: genome-wide profiling of CRISPR-Cas9 off-target effects in human cells. *Nat Methods* **12**, 237–243 (2015).
5. Kim, D., Kim, S., Kim, S., Park, J. & Kim, J. S. Genome-wide target specificities of CRISPR-Cas9 nucleases revealed by multiplex Digenome-seq. *Genome Res.* **26**, 406–415 (2016).
6. Tsai, S. Q. *et al.* GUIDE-seq enables genome-wide profiling of off-target cleavage by CRISPR-Cas nucleases. *Nat. Biotechnol.* **33**, 187–198 (2015).
7. Frock, R. L. *et al.* Genome-wide detection of DNA double-stranded breaks induced by engineered nucleases. *Nat. Biotechnol.* **33**, 179–188 (2015).
8. Cameron, P. *et al.* Mapping the genomic landscape of CRISPR-Cas9 cleavage. *Nat. Methods* **14**, 600–606 (2017).
