# Supplementary material for: A kinetic model predicts SpCas9 activity, improves off-target classification, and reveals the physical basis of targeting fidelity
Source: Nat Commun. 2022 Mar 15;13:1367. doi: 10.1038/s41467-022-28994-2 (PMC8924176; doi:10.1038/s41467-022-28994-2)
Supplement: Supplementary file 3 — Description of Additional Supplementary Files [file 41467_2022_28994_MOESM3_ESM.pdf]

Description of Additional Supplementary Files

File Name: Supplementary Movie 1
Description: The video shows the 33 near equivalent optimization runs (residue within 15% of the optimal run) with Monte Carlo steps mapped to time. To the top left are the estimated on-target free-energy landscapes, and to the top right are the estimated mismatch penalties. At the bottom left, the estimated single-mismatch effective cleavage rate (line) compared to the mismatch-type averaged cleavage rate measured in NucleSeq experiments (triangles). At the bottom right, the predicted effective association constant (line) compared to the mismatch-type averaged effective association constant measured in CHAMP experiments (triangles). The fit is performed by optimizing over both single- and double- mismatched targets.

File Name: Supplementary Data 1
Description: This file contains raw data used for figures.
Mismatch type averaged data used for training and validation in Figure 2 and Figure 3 a,b.
Mismatch type averaged data used for validation in Figure 3c-h.
Optimal microscopic parameters displayed as pink data points in Figure 4.
Result of genome wide analysis underlying Figure 7b-e.
